# Abdominal pain: ingestion of a toothpick leads to a presentation similar to acute pancreatitis

**DOI:** 10.1055/a-2413-7824

**Published:** 2024-10-25

**Authors:** SaiLing Wei, JiaXun Xie, Huan He, HaiTao Zhang, Muhan Lü, Lei Shi

**Affiliations:** 1 556508 Gastroenterology, The Affiliated Hospital of Southwest Medical University, Luzhou, China

A 39-year-old man presented to our hospital with abdominal pain for 12 days. His serum amylase concentration was 772.4 U/L and his pancreatic amylase concentration was 529.9 U/L. Abdominal computed tomography (CT) demonstrated a thin strip of high density shadowing on the lower curved side of the stomach body and the pancreatic body, consistent with pancreatitis ( Fig. 1 ). After a detailed medical history had been taken, the patient could not remember whether he had ingested any foreign bodies.

**Fig. 1:**
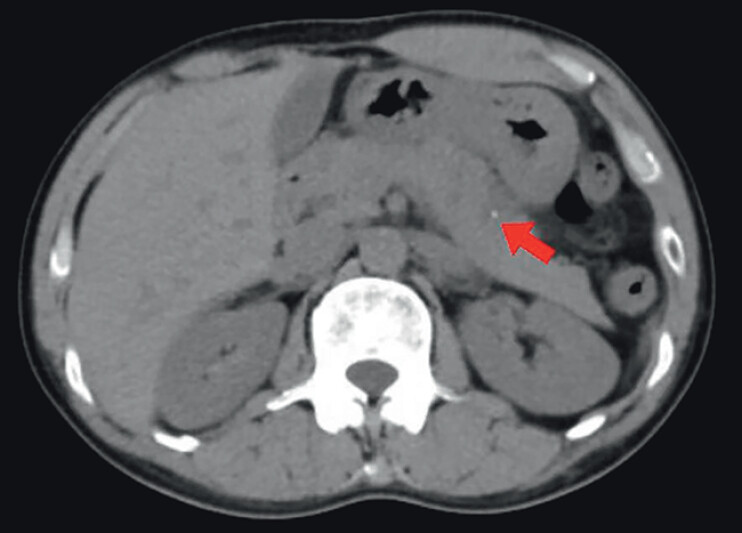
Abdominal computed tomography image showing a thin strip of high density shadowing on the lower curved side of the stomach body and the pancreatic body.

Gastroscopy revealed an elevated lesion on the upper lesser curvature of the stomach, with a small amount of purulent secretion attached ( Fig. 2 ). Endoscopic ultrasound (EUS) revealed a strong echo signal, with a length of about 35 mm, in the eminence of the stomach. One end was located superficially in the submucosa, and the other end had penetrated the stomach wall and was inserted into the pancreas ( Fig. 3 ). Based on these examinations, we chose to perform endoscopic foreign body removal ( Video 1 ). Because the EUS had suggested that one end of the foreign body was superficially located in the submucosa, we looked to directly explore the foreign body by clamping the breach with a foreign body forceps. The toothpick was revealed and successfully removed ( Fig. 4 ). The patient’s abdominal pain was relieved and his amylase had decreased to a normal level 1 day after the surgery.

**Fig. 2:**
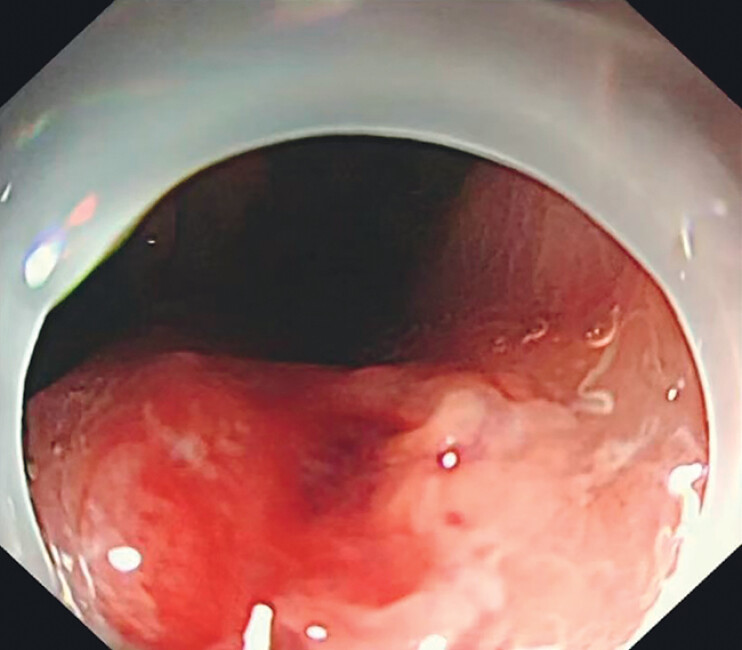
Gastroscopic image showing an elevated lesion on the upper lesser curvature of the stomach, with a small amount of purulent secretions attached.

**Fig. 3:**
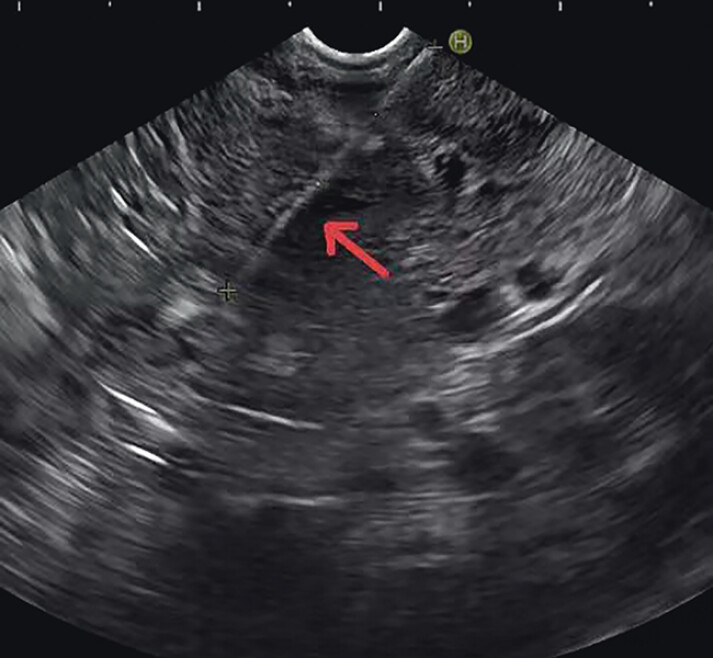
Endoscopic ultrasound image showing a strong echo signal in the eminence of the stomach, with one end located superficially in the submucosa and the other end penetrating the stomach wall and inserting into the pancreas.

A toothpick that had penetrated the stomach wall and inserted into the pancreas causing abdominal pain and blood changes consistent with pancreatitis is identified on endoscopic ultrasound and removed with foreign body forceps.Video 1

**Fig. 4:**
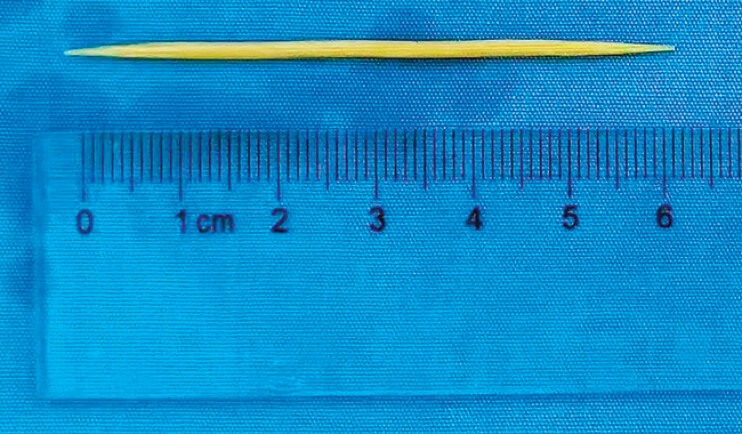
Photograph of the extracted toothpick.

It is rare for a foreign body to penetrate the stomach wall and migrate to the pancreas 1 . There have been previous case reports of removal of this type of foreign body by open or laparoscopic surgery 2 3 . In this case, with the information provided by the EUS, the toothpick was successfully removed endoscopically, and the patientʼs symptoms were relieved, with the pancreatic tests returning to normal after the operation. This report shows that endoscopic removal of foreign bodies that have penetrated the pancreas is safe and feasible 4 .

Endoscopy_UCTN_Code_TTT_1AO_2AL

## Notes

Endoscopy E-Videos https://eref.thieme.de/e-videos
*E-Videos* is an open access online section of the journal *Endoscopy* , reporting on interesting cases and new techniques in gastroenterological endoscopy. All papers include a high-quality video and are published with a Creative Commons CC-BY license. Endoscopy E-Videos qualify for HINARI discounts and waivers and eligibility is automatically checked during the submission process. We grant 100% waivers to articles whose corresponding authors are based in Group A countries and 50% waivers to those who are based in Group B countries as classified by Research4Life (see: https://www.research4life.org/access/eligibility/ ). This section has its own submission website at https://mc.manuscriptcentral.com/e-videos .
